# A reimbursement framework for artificial intelligence in healthcare

**DOI:** 10.1038/s41746-022-00621-w

**Published:** 2022-06-09

**Authors:** Michael D. Abràmoff, Cybil Roehrenbeck, Sylvia Trujillo, Juli Goldstein, Anitra S. Graves, Michael X. Repka, Ezequiel “Zeke” Silva III

**Affiliations:** 1 Department of Ophthalmology and Visual Sciences, University of Iowa, Iowa City, IA USA; 2 AI Healthcare Coalition, Washington, DC USA; 3 Digital Diagnostics, Coralville, IA USA; 4 Hogan Lovells LLP, Washington, DC USA; 5 OCHIN, Portland, OR USA; 6 Indiana University School of Medicine, Vincennes, IN USA; 7 Wilmer Eye Institute, Johns Hopkins University, Baltimore, MD USA; 8 South Texas Radiology, San Antonio, TX USA; 9 University of Texas Health, Long School of Medicine, San Antonio, TX USA

**Keywords:** Health services, Translational research

## Abstract

Responsible adoption of healthcare artificial intelligence (AI) requires that AI systems which benefit patients and populations, including autonomous AI systems, are incentivized financially at a consistent and sustainable level. We present a framework for analytically determining value and cost of each unique AI service. The framework’s processes involve affected stakeholders, including patients, providers, legislators, payors, and AI creators, in order to find an optimum balance among ethics, workflow, cost, and value as identified by each of these stakeholders. We use a real world, completed, an example of a specific autonomous AI service, to show how multiple “guardrails” for the AI system implementation enforce ethical principles. It can guide the development of sustainable reimbursement for future AI services, ensuring the quality of care, healthcare equity, and mitigation of potential bias, and thereby contribute to realize the potential of AI to improve clinical outcomes for patients and populations, improve access, remove disparities, and reduce cost.

## Introduction

Responsible adoption of healthcare artificial intelligence (AI) requires that AI systems that benefit patients and populations are incentivized financially at a consistent and sustainable level. At the same time, AI that does not offer such health benefits should not be incentivized. We focus on patient-specific, assistive, and autonomous AI systems, where such financial incentives are overseen by both public and private payors, while also requiring involvement, oversight, and support by affected stakeholders in healthcare, including patients, policy makers, clinicians, regulators, provider organizations, bioethicists, and AI creators. Such support and involvement were solicited as documented in early studies by Abramoff and other researchers, which led to an ethical framework for healthcare AI^1,2^.

Herein, we use the definition of AI systems used elsewhere:^1^ systems that learn from training data in order to, when deployed, perform tasks that are intended to mimic or extend human cognitive capabilities. These include highly cognitive tasks, such as those typically performed by trained healthcare professionals, and are not explicitly programmed.

AI has now entered mainstream healthcare. This is illustrated by the U.S. Centers for Medicare and Medicaid Services (CMS), for the first time, establishing a national payment amount for an FDA *de novo* authorized autonomous AI system, in both the Medicare Physician Fee Schedule (MPFS), and the Outpatient Prospective Payment System (OPPS), for IDx-DR system (Digital Diagnostics, Coralville, Iowa, USA)^3^. This autonomous^4^ AI system makes a clinical decision without human oversight, and diagnoses a specific disease, as described by its new CPT® code 92229. CMS also, for the first time, established a national add-on payment for assistive AI, under the New Technology Add-on Payments (NTAP) in the Inpatient Prospective Payment System (IPPS), for the Viz LVO system for stroke detection^5^, and the Caption Guidance system for cardiac ultrasound^6^, to enable expanded access.

Now, more than ever before, AI designed, developed, validated, and deployed to promote safety and efficacy is poised to realize the aforementioned advantages. Thereby, it can address the escalation in cost, lack of access to essential healthcare services, and the resulting injustice of health disparities, as well as to advance health equity^7,8^, and improved clinical outcomes. While adoption of AI is growing, so is the evidence for a positive impact created by FDA-regulated AI systems^9,10^, though there is also evidence for negative impact, particularly with unregulated AI systems^9,11,12^.

The decision by providers and health care systems on whether to adopt and deploy a specific healthcare AI is greatly influenced by payment amounts. From their perspective, financial incentives for a service are determined both by *reimbursemen*t, the dollar amount paid for the service, as well as *coverage*, the likelihood of payment when the service is provided to a specific patient for a medically indicated reason. While it is beyond the scope of this manuscript, the issue of medical liability also plays a role, and for autonomous AI, the American Medical Association’s Augmented Intelligence Policy requires autonomous AI creators to assume liability for their performance^13^.

An existing payment framework that has been implemented is the NTAP mentioned above^6^. While it has led to AI payments by CMS, at the moment of writing it is an extremely high bar to reach: it is technology-specific with a complicated approval pathway. Currently, NTAP is limited to services provided to inpatients, as part of IPPS, and does not cover outpatient or physician care; payments are time-limited as it lasts only 3 years for a specific technology or specific indication; the maximum payment is limited to 65% of the cost, and only if a hospital’s costs for a specific stay exceed the MS-DRG bundled payment amount, which means the hospital has to show financial loss; finally, it requires ‘newness’, cost, and substantial clinical improvement criteria for the technology, which are difficult to satisfy—though FDA breakthrough status for new technology can satisfy newness and clinical improvement criteria, newness may only last until utilization claims data exists. Other technologies may become eligible for the NTAP in-patient should they satisfy the very stringent requirements for the add-on payment, as viz.ai and Caption Health have already been able to achieve.

Given the above limitations, others have tried to develop incentive frameworks for AI but only in a limited context, without considering existing, complex multi-payor US healthcare coverage and reimbursement systems, and ignoring the role of affected stakeholders^14^.

Instead, we propose a comprehensive framework that: (a) maximizes alignment with ethical frameworks for healthcare AI^1,2^, (b) allows more optimal alignment between the ethical, equity, workflow, cost, and value perspectives for AI services, (c) enhances support and involvement by affected stakeholders, (d) is transparent, and (e) maps onto the existing payment amount and coverage systems in the US. Its goal is to quantify and estimate the payment amount, which can be optimized by finding an optimum for the framework. One such optimum might be where it cannot be improved for some stakeholders, without making it less optimal for others, called a Pareto optimum^15^.

While we focus on the US landscape, this framework can be a useful template for any healthcare system. The framework is focused on payment for the use of AI, and while implementing an AI may have downstream effects, such as more patients being recommended for treatment, or a provider needing a more or less complex E/M visit, is beyond its scope, as there are existing methodologies and frameworks to adjust payments for such cases if appropriate. As a practical example, we provide a case study to demonstrate how this framework, involving affected stakeholders, can lead to insurance coverage and sustainable reimbursement for a specific validated autonomous AI service.

Finally, we alert the reader that the first author is an AI creator, and therefore is potentially biased towards monetary reimbursement for AI; a similar conflict exists for JG. While all potential conflicts have been fully disclosed, we have sought to ensure a balanced discussion as the other authors are not conflicted, are on the professional and payor side, and do not have financial or other interest in any AI receiving monetary reimbursement.

### Concerns about healthcare AI

As mentioned, healthcare can negatively affect outcomes and undesirable effects of the rapid introduction of AI into clinical practice have been described^1,16,17^, including:

- Whether AI improves patient and population clinical outcomes (rather than worsening them); and
- AI bias and impact on health equity; and
- Potential lack of data privacy, meaningful consent, stewardship responsibilities, and ownership; and
- How liability is assigned.

While a comprehensive review of such valid and real concerns with healthcare AI, as well as their interaction is beyond the scope of this comment, we want to give a recently published example. Our goal is to alert the reader that a basis in an ethical framework can be helpful to discover, prevent, or mitigate other ethical concerns with healthcare AI. We clarify that this Comment is not meant to be an introduction to AI ethics, or the metrics used to evaluate AI ethics. We refer instead to previous publications on AI ethics, where terms such as sensitivity, specificity, and equity are discussed in detail^1,2^. One study found racial bias in a widely used AI, which assigned Black patients the same level of risk of a poor clinical outcome even though they were sicker than White patients, so that the number of Black patients identified for extra care was halved^18^. Consequently, as we consider a framework for appropriate reimbursement of healthcare AI, it is important to incentivize AI systems that have been clinically validated under FDA oversight or otherwise, and align with an ethical framework.

### Considerations of financial incentives for healthcare AI

As mentioned, whether or not to deploy or use a specific healthcare AI is influenced greatly by payment and coverage policies, for digital technology^19^. This is illustrated by the recent expansion of telemedicine during the COVID-19 public health emergency, where coverage and reimbursement were available under a broader range of conditions^20^. We propose that a sustainable and optimal balance between value and cost will be crucial for the successful adoption and integration of healthcare AI.

As we consider payment amounts, as well as the complexity of our healthcare system, and the interdependencies between all the stakeholders, it follows that a high degree of alignment between stakeholders is essential. While different healthcare stakeholders necessarily weigh specific benefits and risks for healthcare AI as more or less important, generally, stakeholders prefer AI that is affordable, high quality, equitable, safe, efficacious, outcome improving, and ethically designed^1^. Meanwhile, creators and investors give substantial weight to sustainable and predictable financial return on their investment—in other words, after design, research and development, validation, and marketing have been paid and accounted for.

### Considerations for the cost and value of AI systems

Few frameworks for the valuation of (autonomous) AI in healthcare exist—and if any exist, they are ad hoc. From a health economics standpoint, we define the cost of AI systems as what AI creators charge for the patient-specific service that uses AI system, for recoupment of their investment in research, development and validation, ongoing operating expenses, liability protection, and assurance of continued patient safety, efficacy, and equity (including the cost of maintenance and updates).

We define the *value* of AI systems—as it were, of the *AI work -*as what payors, patients, providers, and society as a whole see as the utility of the service in terms of improved clinical outcomes—at both patient and population levels, access to care, and provider satisfaction, in addition to any downstream cost savings^9^. CMS and others use a shorthand for this same definition: *value* = *quality/cost*. In this relationship for diagnostic services, value goes up when diagnostic accuracy is better and if cost is less, other factors being equal^21^. We next consider a non-exhaustive set of approaches to derive the value of a given service involving AI:

- The *cost-effective value* is derived from cost benefit analyses (CBA) or cost effectiveness analyses (CEA). These models analyze the extra expenditures if a service is not provided, compared to when the service is provided. Such analyses are based on many assumptions, especially about the value people place on their health^22^. For example, a late diagnosis increases treatment costs, or a poorer outcome has adverse financial consequences. The cost benefit threshold is where expenditures for the service equals the extra expenditures across the at-risk population.
- The *substitution value*, derived from what payors are currently paying for the service provided by a—human—provider, such as specialist. Many of these services are valued based on the provider’s expertize, time spent, and ancillary services required.
- The *access maximizing value*, derived from the current total value for a subset of the population assigned to the entire population getting the service, at the population level:

Let *e*_*c*_ be the current total expenditure for a service, performed by humans only; *v*_*c*_, the current value per patient that undergoes that service; *n* the number of patients at risk who would benefit from that service; and *c* the fraction of *n* that undergoes that service—the ‘compliant’ population—, then

$$e_c = v_cnc$$

*e*_*c*_ is the lower bound of “payor willingness to pay,” in other words, the rate at which payors like CMS are currently reimbursing the service, without AI, for Medicare beneficiaries. The assumptions are a) “payor willingness to pay” does not go down when the entire population undergoes the service, instead of just a subset, and b) that the AI can service the entire at-risk population *n*. Thus, when *e* is the total expenditure at full access, then

$$e = v_en = e_c;\,{{{\mathrm{or}}}},\,v_e = v_cc$$

with *v*_*e*_ the lower bound for the *access maximizing value* per patient, when using AI. In effect, the total expenditure is capped at the “payor willingness to pay,” with *v*_*e*_ potentially allowing all patients to undergo the service.

We can also consider the cost to provide a service to use as a starting point for the value of a given service involving AI, as follows:

- *Marginal cost*. One of the advantages of AI is its scalability. Marginal cost considers the cost of a single patient’s diagnostic service, i.e., the incremental cost for one more patient, after the AI has already diagnosed (for example) a million patients. The marginal cost disregards necessary investment in R&D, including training the AI^23^, validation of safety, and efficacy^1^, even though ongoing monitoring and quality assurance will be required. Marginal cost is then the sum of the cost of continuing to operate the AI in the workflow, patient specific clinical labor, supplies and equipment, liability protections, electricity, and other resources for running the AI software for that patient. The marginal cost of the pure AI decision itself—mostly inference—is typically low^23^.
- *Total cost of ownership*, reflects the sum of investment in R&D, including training the AI, validation of safety, and efficacy, as well as the ongoing marginal costs mentioned above. Given these high upfront costs for AI, charging the total cost of ownership, such as a capital expense model, is likely only affordable to the wealthiest healthcare systems. Under-resourced healthcare systems and providers will not be able to afford the AI, leading to diminished access and potentially increasing healthcare disparities.

Considering these alternatives to derive the value of an AI service, and considering the goals of different stakeholders, we can see that, using the *cost-effective value* or the *substitution value* are unlikely to lead to any cost savings, and in fact, the *cost-effective value* may not be optimal^15^. Using the *total cost of ownership* is likely to increase health disparities rather than decrease them. Using *marginal cost* will not be sustainable for AI creators.

Under this framework, the *access maximizing value v*_*e*_, decreasing expenditure per patient, and simultaneously incentivizing access, is an attractive payment derivation. Understanding the economic value of an AI service in healthcare is required for AI creators, as the AI’s economic value forms an important constraint during the AI product development lifecycle, in addition to the constraints derived from ethical considerations, such as high accuracy, efficacy and lack of bias, and requirements derived from workflow needs such as autonomy, clinical labor requirements, availability at point-of-care, liability considerations, and user experience.

### Practical implementation of establishing values for services that include an autonomous AI

Analyzing reimbursement on a per patient cost, as per the above framework, additionally aligns to the model of relative valuation within the Medicare Physician Fee Schedule and its Resource Based Relative Value Scale (RBRVS), which includes distinct physician work, professional liability, and practice expense relative value units (RVUs)^24^. Practice expense within the RBRVS are considered either “direct” or “indirect”. Direct expenses are priced by CMS based on invoices charged in a competitive marketplace by suppliers of the AI service^25^. This allows the AI system value to be mapped onto the existing regulatory and reimbursement structures within practice expense. In fact, stakeholders have at their disposal existing tools to ensure AI systems are aligned with their specific goals, such as:

- Federal legislative and Executive branches of the US government: for example, federal legislation that impacts the adoption of healthcare AI, such as the 21st Century Cures Act and related amendments to the Social Security Act, and also the formation of the National Artificial Intelligence Advisory Committee (NAIAC) in the Executive Branch^26^.
- Regulators: the US Food and Drug Administration (FDA) has developed and continues to develop a wide range of regulatory guidelines and discussion papers on AI systems (termed Software as a Medical Device or SaMD) safety^27–30^, and ethics, in collaboration with other stakeholders^1,24^. The Federal Trade Commission has regulatory oversight, but does not ensure the safety, and efficacy, of products. Finally, the U.S. Department of Health and Human Services’ Office of Civil Rights, and state consumer protection and civil rights agencies, may take action against discriminatory practices due to AI systems.
- Patients and patient organizations: creation and updating of Standards of Care, such as ADA’s Standards of Medical Care for Diabetes and updating it to support the use of autonomous AI for the diabetic retinal exam^31^, involvement in the comment process for CMS proposed rules, and in FDA’s regulatory processes through the Patient Preference Initiative and other guidance^32^
- Evidence-based medicine experts: the National Committee of Quality Assurance (NCQA) can create or update new quality measures, described as the Healthcare Effectiveness Data and Information Set (HEDIS), and there are financial incentives tied to meeting a HEDIS measure for payors and providers. The United States Preventative Services Task Force (USPSTF) makes evidence-based recommendations about preventative services, which may include AI, which can affect patient access.
- Physician and provider organizations: the American Medical Association’s Current Procedural Terminology (CPT®) Editorial Panel evaluates new services, including digital technology and those supported by AI systems, through literature analysis, determination of widespread use and FDA regulatory authorization, evaluating whether they meet criteria of safety, effectiveness, and usefulness; and subsequently creates or updates CPT® codes^33^. The CPT Editorial Panel has published a taxonomy describing a hierarchy of AI services^34^. The RVS Update Committee (RUC) makes recommendations on the physician work and direct practice expenses of the AI services to CMS^35^. Where applicable, whether the patient continues to have a “medical home” when AI systems are implemented may be important.
- Government insurance: the Centers for Medicare and Medicaid Services (CMS) periodically creates coverage and reimbursement, through its rule making processes, and CMS considers practice expense, market-based supply, and equipment pricing updates, as well as rate-setting refinements, malpractice expense, labor rates, and geographic practice cost indices to set rates for the Medicare Physician Fee Schedule (MPFS)^25,36^, Outpatient Prospective Payment System (OPPS), and Inpatient Prospective Payment System (IPPS)^37^. Additionally, CMS administers the Merit-based Incentive Payment System (MIPS), which requires payment adjustments according to performance on cost and quality measurements for certain eligible Medicare Part B providers. MIPS measures may also align with external quality measure sets, such as HEDIS, and thereby can affect services using AI.
- Commercial health insurance: these entities have their own processes to setting their rate; in practice they often set reimbursement by applying a >100% percentage of Medicare PFS^38^.

Collectively, the processes described above on which stakeholders engage are important first steps in creating a complete system of guardrails exist. The goal of these guardrails is to address concerns how a specific healthcare AI system affects patients and populations with respect to clinical outcomes, safety, AI bias, cost, and health equity.

As presented, the above, transparent, framework allows optimizing a balance between the ethical, workflow, cost, and value perspectives on AI services, by all stakeholders^15^. An earlier form of this framework was presented by the first author as part of a US Congressional briefing on May 28, 2019^39^.

### Real-world case study: Autonomous AI for the diabetic retinal exam

In this real-world example, we examine how the framework can be used for an autonomous AI for the diabetic retinal exam that diagnoses diabetic retinopathy and diabetic macular edema without human oversight. This AI system (IDx-DR, Digital Diagnostics Inc, Coralville, IA), is used in primary care and endocrinology clinics where patients with diabetes are managed. Rather than a patient being referred to an eye care provider for the diabetic retinal exam, the autonomous AI system, which includes a robotic fundus camera, makes a point of care diagnosis in real time, and an individualized diagnostic report without review by a human. Only if the result is abnormal, i.e., disease is present, is the patient referred to an eye care provider. It received *de novo* FDA authorization in April 2018^40^, after extensive clinical testing^41^, and has since been widely implemented. We examine both how the AI creator came to the cost for the use of the AI system, and how all stakeholders were involved in creating the guardrails and appropriate payment for the use of the AI system.

### Analyzing AI system cost, under an access maximizing value approach

The creator used the *access maximizing value* approach to arrive at *v*_*e*_ ≅ 55, based on the following assumptions: *v*_*c*_ = $175 for the Medicare population, based on the median Medicare reimbursement for an eye exam by an eye care provider in 2015;^36^ *c* = 0.3 for the Medicare population—the estimated compliance with the diabetic retinal exam in that population^42,43^. This *v*_*e*_ thus served as an anchor for the AI creator to charge $55 per patient for this AI service, the AI work, as well as the cost to obtain inputs and manage AI output. Under this approach, while AI creators can use the per patient *v*_*e*_, or any other value or cost, as a design anchor, this does not preclude them charging for the AI service instead, per volume, per population, or per usage. This allowed the creator to optimize for this cost; for example, choosing a more expensive retinal camera hardware might allow higher accuracy and diagnosability at lower R&D expenses for the diagnostic artificial intelligence algorithms, but would increase the cost per patient to a level that exceeds *v*_*e*_. A less expensive camera instead required a more sophisticated AI algorithms, with higher R&D expenses, but because of AI’s scalability, allows the creator to still meet the *v*_*e*_ chosen above.

### Reimbursement and guardrails to autonomous AI for the diabetic retinal exam

The guardrails allow continuous and ongoing focus on support by all stakeholders, and they allowed a specific AI system for the autonomous AI diabetic retinal exam, supported by all stakeholders, and its reimbursement, to be determined:

- Regulators: As mentioned, the pivotal trial for this autonomous AI system was completed in 2017, and because the endpoints included safety, equity, and efficacy, as well as addressing bias^41^, FDA *De Novo* authorized this autonomous AI in 2018^40^. Specifically, this preregistered pivotal trial included hypothesis testing for the statistical absence or presence of racial, ethnic, and sex bias on diagnostic sensitivity and specificity, and which was indeed confirmed. As a prescription device, it ensures the patient remains in the medical home.
- Patient and patient organizations: The scientific evidence for the safety and efficacy of this autonomous AI was cited by the American Diabetes Association in the 2020 update to its Standards of Medical Care for Diabetes. This updated standard of care supported the use of autonomous AI (for the diabetic retinal exam) for the first time^31^.
- Evidence-based medicine experts: NCQA updated HEDIS measurement language to support the use of autonomous AI to close the diabetic retinal exam care gap and meet this measure^44^. It measures the percentage of people with diabetes in a covered population that has received a documented diabetic retinal exam, and intends to incentivize payors and provider organizations to have this measure be at least 80%^45^. USPSTF confirmed that the diabetic retinal exam, is not a primary preventative service, as the patient has been diagnosed with diabetes (personal communication).
- Physician and provider organizations: The CPT® Editorial Panel created the first CPT® code for autonomous AI (for the diabetic retinal exam), 92229, in May 2019^46^, and noted: “*The addition of code 92229 for retinal imaging with automated point-of-care, […], increase early detection and incorporation of findings into diabetes care. Innovative solutions like the augmented intelligence technology described by new code 92229 have the potential to improve access for at-risk patient populations by bringing retinal imaging capabilities into the primary care setting*”^47^.
- Government health programs: CMS in November 2021 finalized their proposal to establish national values for the autonomous diabetic retinal exam (described by CPT® code 92229) with transparent RVUs at $45–$64 per exam, per Jan 1, 2022^48^. As usual, the exact reimbursement per patient that a provider using the AI service will receive depends on geographic and other factors. CMS also confirmed that the autonomous AI exam qualifies for MIPS measure 117, and can close the diabetic retinal exam care gap^49^.
- Commercial insurance: as mentioned, these entities refer to CMS rate setting as well as other factors, to establish their own payment amounts.

These decisions have laid the groundwork for the financial sustainability of healthcare autonomous AI for the diabetic retinal exam, resulting in an optimum between the ethical, workflow, cost, and value perspectives on AI services^15^. In addition, this decision allows similar, transparent, analyses of cost and value for other AI services’ reimbursement.

## Discussion

We have presented a framework for establishing reimbursement for the work component of healthcare AI. It aligns with existing ethical frameworks for AI^1^. In fact, it is focused on the enhancement of health equity—more patients receiving the service, especially where currently underserved—reducing cost per patient, and predictable sustainable financial incentives for AI creators. It allows optimality between ethical, workflow, cost, and value, as well as sustainability perspectives on AI services, thus allowing continued support by all stakeholders including patients, providers, legislators, payors, and AI creators, such as the AI Healthcare Coalition (www.ai-coalition.org). We demonstrated how, for a specific autonomous AI service, the diabetic retinal exam, the present framework maps well onto existing reimbursement, regulatory, and value-based care processes, and how sustainable reimbursement can be achieved in a transparent manner. The present framework is designed so that AI systems, that have been shown to be safe, effective, and where potential bias has been mitigated, and developed under an ethical framework, can be priced and reimbursed at a sustainable level, with multiple “guardrails” overseen by all stakeholders that enforce ethical principles overseen by regulators, providers, and patient organizations. The resulting reimbursement allows for sustainable, predictable financial incentives for AI creators, and continued research.

The financial incentive framework presented here may be helpful in analyzing the value and cost of each unique AI service, to guide the development of sustainable reimbursement for future AI services, while ensuring the quality of care, healthcare equity, and where potential bias has been mitigated. Thus, appropriate financial incentives for healthcare AI will contribute to realizing the potential of AI to improve clinical outcomes for patients and populations, remove disparities, lower cost, and improve access.
